# STAT1-dependent expression of energy metabolic pathways links tumour growth and radioresistance to the Warburg effect

**DOI:** 10.1186/1741-7015-7-68

**Published:** 2009-11-05

**Authors:** Sean P Pitroda, Bassam T Wakim, Ravi F Sood, Mara G Beveridge, Michael A Beckett, Dhara M MacDermed, Ralph R Weichselbaum, Nikolai N Khodarev

**Affiliations:** 1 Department of Radiation and Cellular Oncology, The University of Chicago, Chicago, IL 60637, USA; 2 Department of Biochemistry, Medical College of Wisconsin, Milwaukee, WI 53226, USA

## Abstract

**Background:**

The Signal Transducer and Activator of Transcription 1 (STAT1) has traditionally been regarded as a transmitter of interferon signaling and a pro-apoptotic tumour suppressor. Recent data have identified new functions of STAT1 associated with tumourigenesis and resistance to genotoxic stress, including ionizing radiation (IR) and chemotherapy. To investigate the mechanisms contributing to the tumourigenic functions of STAT1, we performed a combined transcriptomic-proteomic expressional analysis and found that STAT1 is associated with regulation of energy metabolism with potential implication in the Warburg effect.

**Methods:**

We generated a stable knockdown of STAT1 in the SCC61 human squamous cell carcinoma cell line, established tumour xenografts in athymic mice, and compared transcriptomic and proteomic profiles of STAT1 wild-type (WT) and knockdown (KD) untreated or irradiated (IR) tumours. Transcriptional profiling was based on Affymetrix Human GeneChip^® ^Gene 1.0 ST microarrays. Proteomes were determined from the tandem mass spectrometry (MS/MS) data by searching against the human subset of the UniProt database. Data were analysed using Significance Analysis of Microarrays for ribonucleic acid and Visualize software for proteins. Functional analysis was performed with Ingenuity Pathway Analysis with statistical significance measured by Fisher's exact test.

**Results:**

Knockdown of STAT1 led to significant growth suppression in untreated tumours and radio sensitization of irradiated tumours. These changes were accompanied by alterations in the expression of genes and proteins of glycolysis/gluconeogenesis (GG), the citrate cycle (CC) and oxidative phosphorylation (OP). Of these pathways, GG had the most concordant changes in gene and protein expression and demonstrated a STAT1-dependent expression of genes and proteins consistent with tumour-specific glycolysis. In addition, IR drastically suppressed the GG pathway in STAT1 KD tumours without significant change in STAT1 WT tumours.

**Conclusion:**

Our results identify a previously uncharacterized function of STAT1 in tumours: expressional regulation of genes encoding proteins involved in glycolysis, the citrate cycle and mitochondrial oxidative phosphorylation, with predominant regulation of glycolytic genes. STAT1-dependent expressional regulation of glycolysis suggests a potential role for STAT1 as a transcriptional modulator of genes responsible for the Warburg effect.

## Background

Signal Transducer and Activator of Transcription 1 (STAT1) is the major transcriptional mediator of interferon (IFN)-induced signaling for Type I (IFNα and IFNβ) and Type II (IFNγ) interferons. While STAT1 has traditionally been regarded as pro-apoptotic and tumour-suppressing [1,2], we previously demonstrated that over-expression of the STAT1 pathway confers radio resistance and IFN-resistance [3-5]. Consistent with our observations are recent reports demonstrating that constitutive over-expression of STAT1 and STAT1-dependent genes is associated with protection of tumour cells from genotoxic stress following treatment with fludarabine [6], doxorubicin [7], cisplatin [8] and the combination of ionizing radiation (IR) and doxorubicin [9,10].

To investigate the mechanisms by which STAT1 confers an aggressive tumour phenotype, we characterized the downstream pathways regulated by STAT1. To this end, we generated a stable STAT1 knockdown (KD) in SCC61, a clinically derived squamous cell carcinoma cell line [3,4], and studied the effect of STAT1 KD on tumour growth and response to IR *in vivo*. We then used a shotgun proteomic approach, coupled with gene array analysis, to identify proteins and genes differentially expressed in wild-type (WT) and KD untreated or irradiated tumours. Our results demonstrate that STAT1 modulates the expression of genes encoding proteins involved in glycolysis/gluconeogenesis (GG), oxidative phosphorylation (OP), and the citrate cycle (CC) and protects against IR-induced suppression of genes and proteins belonging to these pathways. Thus, we report that a previously uncharacterized role of STAT1 in regulating the expression of genes involved in energy metabolism may mediate enhanced tumour growth and radio resistance. Our results are consistent with Warburg's finding that tumour cells utilize glycolysis as the main pathway of energy metabolism even in the presence of oxygen [11] and suggest that STAT1 is involved in the transcriptional regulation of the Warburg effect in tumour cells.

## Methods

### Cell culture and tumour model

The SCC61 human squamous cell carcinoma cell line was stably transfected with a control vector (SCC61 STAT1 WT) or one expressing a short hairpin ribonucleic acid (RNA) to STAT1 (SCC61 STAT1 KD) and maintained as previously described [4]. Tumour xenografts were established by a subcutaneous injection of 10^7 ^cells in 100 μL of phosphate buffered saline into the right hind limbs of 6-week-old female athymic mice (FCRI-Taconic). When tumours reached an average size of 160 mm^3^, ionizing radiation was delivered in 5 Gy fractions over six consecutive days (total 30 Gy) using a Philips RT 250 X-ray generator with a dose rate of 1.65 Gy/min. Tumour volumes of untreated control (C) and IR tumours were determined by direct measurement with calipers and calculated using the formula *volume *= *length *× *width *× *depth*/2.

Tumour data represent the mean tumour volume ± standard error of mean (SEM). Each point summarizes data from four to nine animals (WT/C: 4; KD/C: 5; WT/IR: 7; KD/IR: 9). Given that stable KD of STAT1 in nu61 resulted in tumour growth suppression and radio sensitization [4], we used 1-tailed Student's *t*-tests to test the null hypothesis of equal mean tumour volume between STAT1 WT and KD tumours for untreated and irradiated conditions. When tumour volume reached 1000 mm^3^, mice were euthanized by using CO_2 _followed by cervical dislocation. Tumours were excised, snap-frozen in liquid nitrogen, and stored at -80°C until RNA and protein extraction. All animal experiments were conducted in accordance with institutional guidelines at The University of Chicago.

### Gene expression data collection and analysis

Frozen tumour xenografts were sectioned into pieces approximately 5 mm^3 ^in size and soaked overnight in RNA*later*^®^-ICE solution (Applied Biosystems-Ambion). Samples were spun, washed in RNeasy Lysis Buffer (RLT) buffer (QIAGEN), and homogenized on ice using a mechanical glass-Teflon homogenizer set at 3000 rpm. Subsequent purification was performed using TRIzol reagent (Invitrogen Life Sciences) as previously described [12]. The quality of samples was assessed using gel electrophoresis in 1.8% agarose and spectrophotometry, and samples of high quality were transferred to the University of Chicago Functional Genomics Facility for labelling and hybridization with Affymetrix Human GeneChip^® ^Gene 1.0 ST Arrays according to the manufacturer's protocol. Each array was hybridized with a pooled sample normalized to total RNA and consisting of RNA obtained from three independent tumour xenografts. Retrieved data were filtered using a multistep filtration method, which involves the application of receiver operating characteristic analysis for the estimation of cutoff signal intensity values [13]. Only probe set identifiers (IDs) having gene assignments (annotation date: 21 July 2008; Affymetrix) were used for analysis. Subsequent analysis was based on pair-wise comparisons (WT/C versus KD/C; WT/IR versus KD/IR; WT/IR versus WT/C; KD/IR versus KD/C) of duplicated arrays using Significance Analysis of Microarrays (SAM) [14] version 3.02. Differentially expressed probe set IDs were selected with a delta value that provided a false discovery ratio of 0. A relative gene expression value was calculated by normalization to the median expression value for the gene across samples. Selected probe set IDs were gene annotated and functionally designated using Ingenuity Pathway Analysis (IPA; Ingenuity Systems, Inc). Fisher's exact test was used to estimate the significance of the incidence of different canonical pathways. This method calculates the probability that the association between an experimental gene set and a reference gene set associated with a canonical pathway is due to random chance. A *P*-value ≤ 0.05 was considered statistically significant and indicated a nonrandom enrichment of an experimental dataset by members of a specific pathway.

### Protein expression data collection and analysis

Frozen tumour xenografts were sectioned into pieces approximately 5 mm^3 ^in size and homogenized on ice, as described above, in a solution of 8 M urea, 18.2 mM dithiothreitol and 2% 3-[(3-Cholamidopropyl)dimethylammonio]-1-propanesulfonate (CHAPS). Samples were spun and supernatants were collected. Peptide extraction of pooled samples, normalized to total protein and consisting of protein obtained from three to seven independent tumour xenografts, was performed as previously described [15]. Peptides were subjected to LC/MS/MS (liquid chromatography MS/MS) analysis using a liquid trap quadruple from Thermo-Fisher coupled to a Surveyor high-performance LC system equipped with a Micro AS auto sampler. The instrument was interfaced with an Aquasil, C18 PicoFrit capillary column (75 μm × 10 cm) from New Objective. The mobile phases consisted of 0.1% formic acid containing 5% acetonitrile (A) and 0.1% formic acid in 95% acetonitrile (B), respectively. A 180-min linear gradient was used. The ions eluted from the column were electro-sprayed at a voltage of 1.75 kV. The proteome of each sample was determined from the MS/MS data by searching against the human subset of the UniProt database. Data were epitomized using Visualize software (Dr Brian Halligan, Biotechnology and Bioengineering Center, Medical College of Wisconsin, USA). The false discovery rate for individual peptides was 0.05. Single peptide hits with an *x*-score greater than 2.5 were accepted in each independent run. In each pair-wise comparison, proteins that were not detected in both runs for at least one sample were removed from further analysis. Total ion current (TIC) was used as a means to quantify the relative abundance of specific proteins between different samples. Subsequent analysis was based on pair-wise comparisons (WT/C versus KD/C; WT/IR versus KD/IR; WT/IR versus WT/C; KD/IR versus KD/C) of duplicated runs. Undetected TIC values were replaced by a value equal to half the minimum value across all proteins. A relative protein expression value was calculated by normalization to the median TIC value for the protein across samples. Proteins were annotated and functionally designated using IPA. Fisher's exact test was used to estimate the significance of the incidence of different canonical pathways as described above.

### Combined expressional analysis of overlapping pathways

To identify the pathways that mutually represented differentially expressed genes and proteins, a Fisher's exact test *P*-value ≤ 0.1 was considered significant. To assess the role of STAT1 in the expression of each energy pathway (GG, OP, CC), all of the genes and proteins belonging to the pathway that were differentially expressed in at least one comparative analysis were combined into a pathway set. Genes not present in the set, but corresponding to differentially expressed proteins, were subsequently added to the pathway set. Multiple probe set IDs for a given gene were averaged to obtain a representative expression value for the gene.

## Results

### Knockdown of STAT1 in SCC61 tumour xenografts leads to growth suppression and radio sensitization

**Figure 1:**
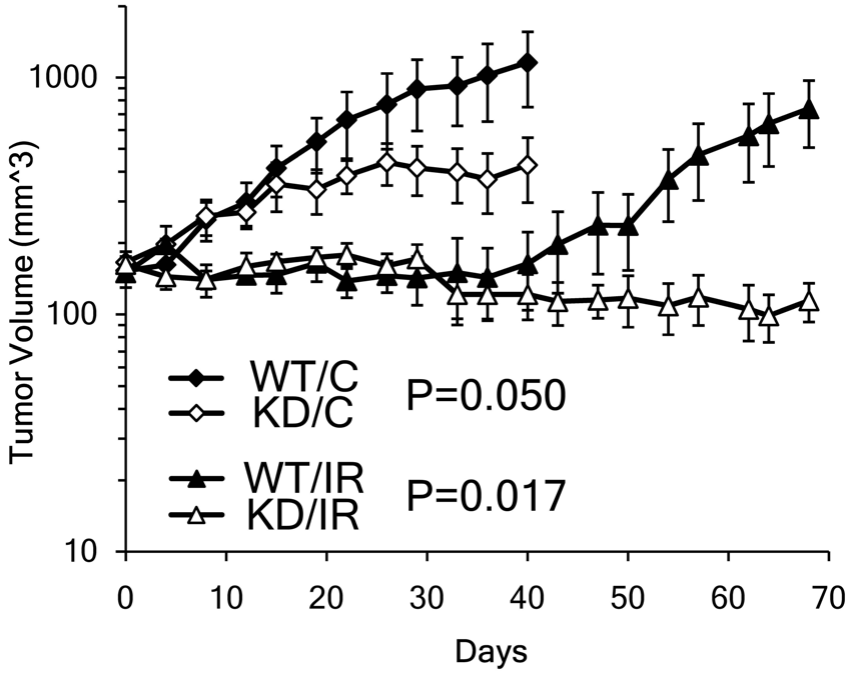
**Knockdown of STAT1 in SCC61 causes tumour growth suppression and radio sensitization**. In untreated tumours, STAT1 [Signal Transducer and Activator of Transcription 1] knockdown (KD/C; white diamond) significantly reduced day 40 tumour volume by 2.7-fold compared to a control-vector transfected STAT1 wild-type (WT/C; black diamond) (mean ± standard error of mean [SEM] [mm^3^]: WT/C = 1154 ± 404, KD/C = 427 ± 130; Student's *t-*test *P *= 0.050). Ionizing radiation (IR) was delivered in 5 Gy fractions on days 0-5 (total 30 Gy). In irradiated tumours, KD of STAT1 (KD/IR; white triangle) significantly suppressed day 68 tumour volume by 6.5-fold compared to that of a STAT1 WT tumour (WT/IR; black triangle) (mean ± SEM [mm^3^]: WT/IR = 738 ± 232, KD/IR = 114 ± 21.2; Student's t-test *P *= 0.017). Point, mean tumour volume; error bars, SEM. Tumour volumes are shown on a semi-log scale.

We previously demonstrated that stable KD of STAT1 in nu61, a radioresistant derivative of the SCC61 tumour cell line, led to decreased tumour growth and sensitization to IR [4]. Here we hypothesized that stable KD of STAT1 in SCC61 would also suppress tumour growth and cause radio sensitization of SCC61. Indeed, KD of STAT1 in untreated tumours led to a significant (*t*-test; *P *= 0.050) 2.7-fold suppression of tumour volume at day 40 relative to STAT1 WT tumours (Figure 1). A comparison of irradiated STAT1 WT and KD tumours demonstrated even larger differences. KD of STAT1 in irradiated tumours significantly (*t-*test; *P *= 0.017) suppressed tumour volume at day 68 by 6.5-fold (Figure 1). These results demonstrate that KD of STAT1 in SCC61, a clinically derived tumour cell line not subjected to any experimental selection, leads to *in vivo *tumour growth suppression and radio sensitization similar to that observed previously in nu61.

### Identification of downstream functions of STAT1 using combined transcriptomic-proteomic expressional analysis

**Figure 2:**
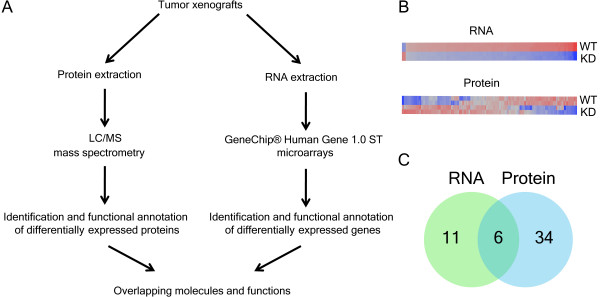
**Combined transcriptomic-proteomic analysis for identification of STAT1-dependent functions**. (A) Outline of transcriptomic-proteomic approach coupled with functional analysis for the identification of STAT1 [Signal Transducer and Activator of Transcription 1]-dependent molecules and functions. (B) Expressional clustering of differentially expressed genes (92) and proteins (266) in untreated SCC61 STAT1 wild-type and knockdown tumours: red, up-regulated; blue, down-regulated. See Additional files 1 and 2 for further details. (C) Venn diagram of all significantly represented pathways on both the transcriptional and translational level. Eleven pathways were uniquely represented among differentially expressed genes, while 34 pathways were uniquely represented among differentially expressed proteins. Six pathways were jointly represented among differentially expressed molecules, and four of these were associated with energy metabolism (see Table 1 for further details).

Using a combination of shotgun proteomics and transcriptional profiling, we investigated the downstream expressional changes associated with KD of STAT1 [16-18]. Protein samples were subjected to LC/MS/MS peptide analysis and RNA samples were hybridized with Affymetrix exon-based arrays (see Methods and GEO accession number GSE15845). Differentially expressed genes and proteins were functionally annotated using IPA as described [19,20] (Figure 2A).

**Table 1:** Pathways mutually represented by differentially expressed Signal Transducer and Activator of Transcription 1 (STAT1)-dependent genes and proteins.

Pathway	*P*-value* (gene)	*P*-value* (protein)
Oxidative phosphorylation	0.00251	0.0955

Citrate cycle	0.00741	0.0182

Urea cycle and metabolism of amino groups	0.00794	0.0224

Pyruvate metabolism	0.0380	0.000151

Nitric oxide signaling in the cardiovascular system	0.0389	0.0245

Glycolysis/gluconeogenesis	0.0589	0.000000000776

*P-value, Fisher's exact test

We compared gene and protein expression profiles of untreated STAT1 WT and KD tumours and identified the differential expression of 92 genes and 266 proteins (Figure 2B; Additional files 1 and 2). Functional analysis of differentially expressed genes and proteins identified a significant overlap between transcriptomic and proteomic approaches. We compared all significantly represented pathways (see Methods) from gene and protein expression patterns and identified six overlapping pathways (Figure 2C; Table 1). Notably, four of the six overlapping pathways (OP, CC, pyruvate metabolism and GG) were associated with energy metabolism. These results show that functional mapping of transcriptomic and proteomic data identifies genes and proteins associated with overlapping functions, which is consistent with other reports [21]. These results also demonstrate that growth suppression of SCC61 tumours caused by KD of STAT1 (see Figure 1) is associated with alterations in the expression of genes and proteins involved in energy metabolism (see Table 1).

### STAT1 modulates the expression of genes encoding proteins involved in glycolysis, oxidative phosphorylation and the citrate cycle

To characterize the role of STAT1 in the expressional regulation of energy metabolic pathways, we examined the expression of genes and proteins involved in GG, the CC and OP because these pathways were jointly represented in both the transcriptomic and proteomic analysis (see Table 1). We performed pair-wise comparisons of STAT1 WT and KD tumours for untreated and irradiated conditions and identified differentially expressed genes and proteins belonging to the GG, CC and OP pathways. We compiled differentially expressed genes and proteins (see Methods; Additional file 3) to identify gene-protein pairs regulated by STAT1 in the same manner (either up- or down-regulated). This analysis identified 22 enzymes that were similarly regulated at the gene and protein level by STAT1 in at least one comparative analysis (Table 2). Sixteen of the 22 gene-protein pairs (72.7%) were associated with GG. The two CC enzymes identified in this analysis and up-regulated by STAT1 produce reduced forms of the coenzymes nicotinamide adenine dinucleotide (NADH: malate dehydrogenase) and flavin adenine dinucleotide (FADH_2_: succinate dehydrogenase), which are electron donors in OP. In addition, three subunits of the soluble catalytic core of mitochondrial adenosine triphosphate (ATP) synthase (ATP5A1, ATP5B and ATP5O) were up-regulated in STAT1 WT relative to KD tumours. Among the 22 gene-protein pairs, the GG pathway was significantly (2-sided Chi-square test; *P *= 0.030) more represented than the OP and CC pathways. These data demonstrate that STAT1 is associated with regulation of the expression of genes encoding proteins involved in energy metabolic pathways, with the majority of identified molecules involved in glycolysis.

**Table 2:** Enzymes co-regulated at the gene and protein level by Signal Transducer and Activator of Transcription 1 (STAT1)

Pathway	Gene Symbol	Description	Function
GG	ALDH1A1	Aldehyde dehydrogenase 1 family, member A1	Oxidation of acetaldehyde to acetate

GG	ALDH2	Aldehyde dehydrogenase 2 family (mitochondrial)	Oxidation of acetaldehyde to acetate

GG	ALDOA	Aldolase A, fructose-bisphosphate	Cleavage of fructose 1,6-bisphosphate into glyceraldehyde 3-phosphate and dihydroxyacetone phosphate

GG	ENO1	Enolase 1, (alpha)	Dehydration of 2-phosphoglycerate to phosphoenolpyruvate

GG	ENO2	Enolase 2 (gamma, neuronal)	Dehydration of 2-phosphoglycerate to phosphoenolpyruvate

GG	ENO3	Enolase 3 (beta, muscle)	Dehydration of 2-phosphoglycerate to phosphoenolpyruvate

GG	GAPDH	Glyceraldehyde-3-phosphate dehydrogenase	Oxidation and phosphorylation of glyceraldehyde 3-phosphate to 1,3-bisphosphoglycerate with reduction of NAD+ to NADH

GG	GPI	Glucose phosphate isomerase	Conversion of glucose 6-phosphate to fructose 6-phosphate

GG	LDHA	Lactate dehydrogenase A	Reduction of pyruvate to lactate with oxidation of NADH to NAD+

GG	LDHAL6B	Lactate dehydrogenase A-like 6B	Reduction of pyruvate to lactate with oxidation of NADH to NAD+

GG	LDHB	Lactate dehydrogenase B	Reduction of pyruvate to lactate with oxidation of NADH to NAD+

GG	PGAM1	Phosphoglycerate mutase 1 (brain)	Conversion of 3-phosphoglycerate to 2-phosphoglycerate

GG	PGK1	Phosphoglycerate kinase 1	Phosphoryl transfer from 1,3-bisphosphoglycerate to ADP with formation of 3-phosphoglycerate and ATP

GG	PGM1	Phosphoglucomutase 1	Interconversion of glucose 1-phosphate and glucose 6-phosphate

GG	PKM2	Pyruvate kinase type 2, muscle	Phosphoryl transfer from phosphoenolpyruvate to ADP with formation of pyruvate and ATP

GG	TPI1	Triosephosphate isomerase 1	Interconversion of dihydroxyacetone phosphate and glyceraldehyde 3-phosphate

OP	ATP5A1	ATP synthase, H+ transporting, mitochondrial F1 complex, alpha subunit 1, cardiac muscle	Synthesis of ATP from ADP and inorganic phosphate

OP	ATP5O	ATP synthase, H+ transporting, mitochondrial F1 complex, O subunit	Synthesis of ATP from ADP and inorganic phosphate

OP	ATP5B	ATP synthase, H+ transporting, mitochondrial F1 complex, beta polypeptide	Synthesis of ATP from ADP and inorganic phosphate

OP	COX4I1	Cytochrome c oxidase subunit IV isoform 1	Oxidation of cytochrome C with reduction of O2 to H2O

OP/CC	SDHA	Succinate dehydrogenase complex, subunit A, flavoprotein (Fp)	Oxidation of succinate to fumarate with reduction of FAD+ to FADH2

CC	MDH2	Malate dehydrogenase 2, NAD (mitochondrial)	Oxidation of malate to oxaloacetate with reduction of NAD+ to NADH

GG, glycolysis/gluconeogenesis; OP, oxidative phosphorylation; CC, citrate cycle

### STAT1 protects tumours from IR-induced suppression of energy pathways

**Figure 3:**
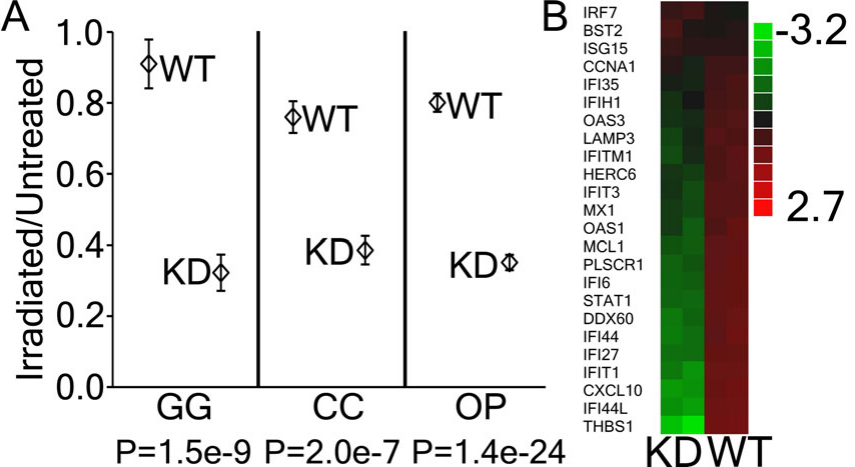
**STAT1 protects tumours from ionizing radiation-induced decreases in the expression of energy metabolic pathways**. (A) Changes in the expression of glycolysis/gluconeogenesis (GG), citrate cycle (CC) and oxidative phosphorylation (OP) energy metabolic pathways in response to ionizing radiation(IR) treatment of STAT1 wild-type (WT) and knockdown (KD) tumours. There was no significant decrease in the expression of the GG pathway in STAT1 WT tumours in the response to IR (mean irradiated/untreated ratio = 0.91; 95% confidence interval [0.77, 1.05]). *P*-values indicate significant differences (Student's 2-tailed *t*-tests) between STAT1 WT and KD tumours. Points, mean value of the ratio of irradiated to untreated for the pathway; error bars, SEM. See Additional file 4 for further details. (B) Expressional clustering of the 24-gene STAT1 pathway in STAT1 KD and WT tumours indicates a significant 3.8-fold up-regulation of the STAT1 pathway (Student's 1-tailed *t*-test; *P *= 7.44e-5). Relative expression values are in log_2 _scale: red, up-regulated; green, down-regulated.

Based on our previous finding, that IR increases the expression of STAT1 and STAT1-dependent genes in several tumour types [4], and our current data, showing that STAT1 regulates the expression of energy metabolic pathways, we hypothesized that STAT1 protects energy metabolic pathways from IR-induced insult. We found that STAT1 WT tumours demonstrated significant protection (*t*-test; *P *< 0.0001) from IR-induced suppression of energy pathways relative to STAT1 KD tumours (Figure 3A and Additional file 4). The GG pathway demonstrated the greatest protection from IR-induced suppression and showed no significant change in STAT1 WT tumours (Figure 3A). Importantly, the STAT1 pathway [3,5] was significantly (*t*-test; *P *= 7.44e-5) up-regulated 3.8-fold in STAT1 WT relative to KD tumours (Figure 3B). These results demonstrate a direct relationship between STAT1 pathway expression and protection of tumour cells from IR-induced suppression of energy pathways and suggest that the survival of irradiated tumour clones (see Figure 1) may be connected with the STAT1-dependent protection of energy pathways.

## Discussion

**Figure 4:**
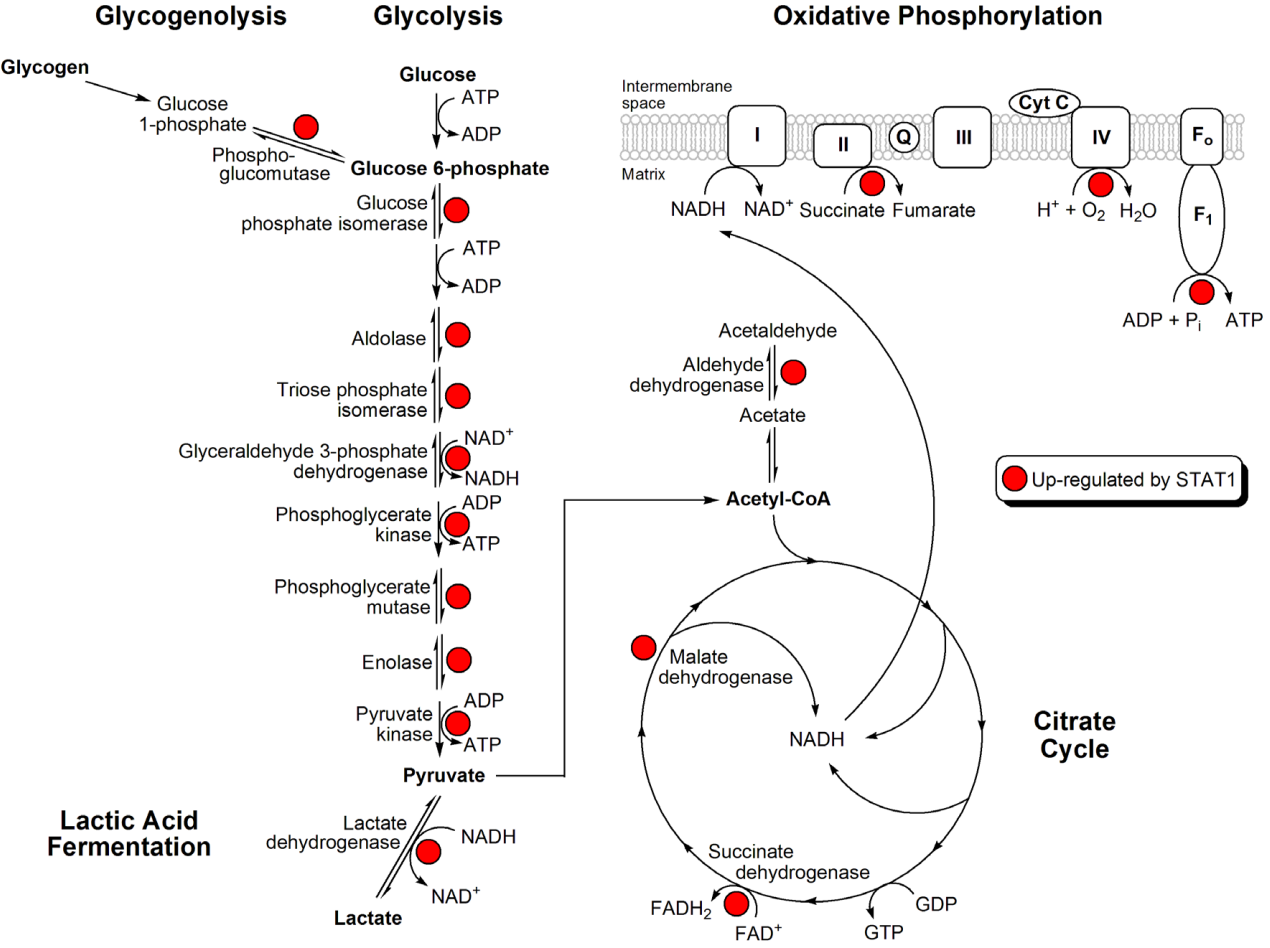
**Energy metabolic map of enzymes up-regulated on the transcriptional and translational levels by STAT1**. The majority of enzymes belonged to the glycolytic pathway. The two citrate cycle enzymes produce reduced forms of the coenzymes nicotinamide adenine dinucleotide (NADH) and flavin adenine dinucleotide (FADH_2_), which donate electrons in the oxidative phosphorylation pathway. Also, three subunits of the catalytic core of mitochondrial adenosine triphosphate synthase (F1) were identified. Red, up-regulated by Signal Transducer and Activator of Transcription 1 (STAT1). See Table 2 for further details.

Utilizing a transcriptomic-proteomic approach together with functional analysis, we report that STAT1 is associated with expression of genes and proteins of energy metabolic pathways including glycolysis, the citrate cycle and oxidative phosphorylation. The STAT1-dependent expression of glycolysis was particularly evident, as 16 enzymes in this pathway were identified in our analysis, with the majority of them up-regulated in STAT1 WT relative to KD tumours (see Table 2 and Figure 4). Given that glycolysis is considered the predominant energy-producing pathway in tumour cells [11] and that high glycolytic flux in tumours has been associated with poor prognosis [22], our data suggest that the tumour-promoting functions of STAT1, described here and reported previously [4], are, at least in part, associated with transcriptional regulation of glycolysis. Further investigation in order to assess the direct metabolic effects of STAT1 on glycolysis will be necessary. Nevertheless, two key enzymes responsible for the maintenance of tumour glycolysis in aerobic conditions - lactate dehydrogenase A (LDHA) [23] and pyruvate kinase type M2 (PKM2) [24] - were among those identified in our analysis as up-regulated by STAT1 at both the gene and protein level (see Table 2 and Figure 4).

These results are consistent with Warburg's finding that glycolysis is the primary mechanism of energy production in tumour cells compared to oxidative phosphorylation [11]. Warburg demonstrated that even in the presence of ample oxygen, tumour cell glycolysis leads to lactate production, similar to that observed in normal cells under anaerobic conditions, without significant involvement of OP (see Figure 4). The molecular mechanisms of this phenomenon, now known as the 'Warburg effect', are currently under extensive investigation. Several oncogenes and tumour suppressor genes have recently been suggested as regulators of the Warburg effect. Among them are TP53 [25], RAS [26], AKT [27], STAT3 [28], and several other candidates (see [29-31] for review). Our results suggest that STAT1-associated transcriptional changes are also involved in regulation of the Warburg effect in tumour cells.

We also report here that ionizing radiation inhibits the expression of genes encoding proteins involved in energy metabolism (see Figure 3). We found that IR treatment resulted in modest suppression of GG, CC and OP gene and protein expression in STAT1 WT tumours. We also reported previously [32] that a 10 Gy dose of IR led to the down-regulation of energy-related genes including LDHA, which is considered a key enzyme of tumour-specific glycolysis [23]. A recent report showed similar IR-induced reductions in the expression of genes related to energy metabolism in human melanoma cells [33]. However, STAT1 KD tumours showed significantly decreased expression of GG, CC and OP genes and proteins relative to WT tumours, indicating that STAT1 is associated with protecting GG, CC and OP expression following IR. Given that STAT1 KD tumours were also significantly radio sensitized compared to WT tumours *in vivo *(Figure 1), our results suggests that a potential mechanism of tumour radio sensitization through STAT1 suppression may be IR-induced energy deprivation of proliferating tumour cells.

Thus, these results identify a previously uncharacterized role of STAT1 in the expressional regulation of genes encoding proteins involved in glycolysis as well as the citrate cycle and oxidative phosphorylation (see Figure 4). In addition, these data demonstrate the ability of STAT1 to protect against IR-induced suppression of genes and proteins belonging to energy-related pathways [32,33] which may protect tumour cells from energy deprivation and mediate radioresistance. Thus, changes in energy-related metabolic pathways may provide growth advantages to tumour cells and support aggressive tumour phenotypes, and STAT1 may be a valuable target for antitumour therapy directed towards energy depletion of proliferating tumour cells.

## Conclusion

We demonstrated that stable KD of STAT1 in the SCC61 human squamous cell carcinoma leads to growth suppression and radio sensitization of tumour xenografts. To define the pathways involved in STAT1-dependent growth and radioresistance, we used a combined transcriptomic-proteomic approach and found that STAT1 is responsible for the expressional regulation of genes and proteins involved in glycolysis, the citrate cycle and oxidative phosphorylation. However, the majority of genes and proteins associated with STAT1 were involved in regulation of glycolysis, a pathway essential to proliferating tumour cells. We conclude that on the transcriptional level, STAT1 contributes to regulation of the Warburg effect, which reflects the dependence of tumour cells on glycolysis as a major pathway of energy production necessary for tumour growth. Our results also demonstrate that STAT1 protects energy pathways from radiation-induced transcriptional suppression, which may in part explain the radio-protective functions of STAT1.

## Abbreviations

ATP: adenosine triphosphate; C: control; CC: citrate cycle; GG: glycolysis/gluconeogenesis; IFN: interferon; IPA: ingenuity pathway analysis; IR: ionizing radiation; KD: knockdown; LC: liquid chromatography; LDHA: lactate dehydrogenase; OP: oxidative phosphorylation; RNA: ribonucleic acid; SAM: significance analysis of microarrays; SEM: standard error of mean; STAT1: Signal Transducer and Activator of Transcription 1; TIC: total ion current; WT: wild-type.

## Competing interests

The authors declare that they have no competing interests.

## Authors' contributions

SPP, RFS, MGB and DMM prepared samples for expressional profiling. BTW performed LC/MS/MS peptide analysis. SPP and BTW acquired gene and protein expression data. MAB conducted animal experiments. SPP, BTW, RFS and NNK analysed and interpreted data. SPP, RFS, NNK and RRW drafted and revised the manuscript. NNK and RRW conceived the study. All authors read and approved the final manuscript. SPP, BTW, and RFS contributed equally. RRW and NNK contributed equally.

## Pre-publication history

The pre-publication history for this paper can be accessed here:

http://www.biomedcentral.com/1741-7015/7/68/prepub

## Supplementary Material

Additional file 1 **Table S1 - Genes differentially expressed between untreated SCC61 Signal Transducer and Activator of Transcription 1 (STAT1) wild-type (WT) and knockdown (KD) tumours**. Probe Set ID = Affymetrix probe set ID; GG = glycolysis/gluconeogenesis; OP = oxidative phosphorylation; CC = citrate cycle; expression values for duplicated SCC61 KD/control (C) and WT/C samples are log_2_-transformed signal intensities normalized to the mean signal intensity across each gene. Click here for file

Additional file 2 **Table S2 - Proteins differentially expressed between untreated SCC61 Signal Transducer and Activator of Transcription 1 (STAT1) wild-type (WT) and knockdown (KD) tumours**. UniProt ID = Universal Protein Resource ID; GG = glycolysis/gluconeogenesis; OP = oxidative phosphorylation; CC = citrate cycle; expression values for duplicated SCC61 KD/C and WT/control (C) samples are log_2_-transformed total ion currents (TIC) normalized to the mean TIC across each protein. Click here for file

Additional file 3 **Table S3 - All differentially expressed genes and proteins associated with energy metabolism from pair-wise comparisons of Signal Transducer and Activator of Transcription 1 (STAT1) wild-type and knockdown tumours for untreated and irradiated conditions**. GG = glycolysis/gluconeogenesis; OP = oxidative phosphorylation; CC = citrate cycle; Probe Set/UniProt ID = Affymetrix probe set/Universal Protein Resource ID; expression values are raw signal intensities for genes or total ion currents for proteins. Click here for file

Additional file 4 **Table S4 - Differential expression of energy metabolic genes and proteins in response to ionizing radiation (IR) of Signal Transducer and Activator of Transcription 1 (STAT1) wild-type (WT) and knockdown (KD) tumours**. GG = glycolysis/gluconeogenesis; OP = oxidative phosphorylation; CC = citrate cycle; (WT/IR)/(WT/C) = response of STAT1 WT tumour to IR; (KD/IR)/(KD/C) = response of STAT1 KD tumour to IR; WT/KD = ratio of WT IR response to KD IR response. Click here for file
